# Supplementary material for: Atmospheric pollution in Ulaanbaatar: Persistence and long-run trends
Source: PLoS One. 2025 Jun 3;20(6):e0322991. doi: 10.1371/journal.pone.0322991 (PMC12132951; doi:10.1371/journal.pone.0322991)
Supplement: Attachment — Submitted filename: FINAL LETTER EXPLAINING THE CHANGES TO THE REVIEWERS.docx [file pone.0322991.s003.docx]

Naranjargal Dashdorj	Prof. Luis Alberiko Gil-Alana

Academic Editor	University of Navarra

PLOS ONE	Pamplona, Spain

email:	[alana@unav.es](mailto:alana@unav.es)

February 27, 2025

**Ms. Ref. No.:** 	PONE-D-24-53171

**Title:**	ATMOSPHERIC POLLUTION IN ULAANBAATAR. PERSISTENCE AND LONG-RUN TRENDS

**Journal:**	PLOS ONE

Dear Editor,

Many thanks for considering our paper entitled “Atmospheric Pollution in Ulaanbaatar. Persistence and Long Run Trends” as a candidate for publication in Plos One. Following your advice and the reviewers’ comments, we have made numerous changes in the paper that we summarize in the following pages. Hoping this time the paper might be acceptable for publication in Plos One,

Yours sincerely,

Prof. Luis Alberiko Gil-Alana

P.S.:	The authors have declared that no competing interests exist.

**Reviewers' comments:**

**Journal Requirements:**

When submitting your revision, we need you to address these additional requirements.

**1.)**	Please ensure that your manuscript meets PLOS ONE's style requirements, including those for file naming. The PLOS ONE style templates can be found at 
<https://journals.plos.org/plosone/s/file?id=wjVg/PLOSOne_formatting_sample_main_body.pdf> and 
<https://journals.plos.org/plosone/s/file?id=ba62/PLOSOne_formatting_sample_title_authors_affiliations.pdf>

**Reply:**	Thanks. We have taken into account the submission guidelines of the journal.

**2.)**	Please include a complete copy of PLOS’ questionnaire on inclusivity in global research in your revised manuscript. Our policy for research in this area aims to improve transparency in the reporting of research performed outside of researchers’ own country or community. The policy applies to researchers who have travelled to a different country to conduct research, research with Indigenous populations or their lands, and research on cultural artefacts. The questionnaire can also be requested at the journal’s discretion for any other submissions, even if these conditions are not met.  Please find more information on the policy and a link to download a blank copy of the questionnaire here: <https://journals.plos.org/plosone/s/best-practices-in-research-reporting>. Please upload a completed version of your questionnaire as Supporting Information when you resubmit your manuscript.

**Reply:** Done.

**3.)**	We noticed you have some minor occurrence of overlapping text with the following previous publication(s), which needs to be addressed:

<https://ddfv.ufv.es/rest/api/core/bitstreams/827ad7ae-0e26-4763-8a14-303d7b4d8968/content>

In your revision ensure you cite all your sources (including your own works), and quote or rephrase any duplicated text outside the methods section. Further consideration is dependent on these concerns being addressed.

**Reply:** Thanks for the comment. The document is another paper of one of the authors of the present manuscript. He employs the same methodology as the one used in this work. That is the reason for the overlapping. We have rewritten some of the paragraphs dealing with the methodology to avoid such similarity.

**4.)**	We note that the grant information you provided in the ‘Funding Information’ and ‘Financial Disclosure’ sections do not match.

When you resubmit, please ensure that you provide the correct grant numbers for the awards you received for your study in the ‘Funding Information’ section.

Corrected. The proper Funding Information is:

Luis A. Gil-Alana gratefully acknowledge financial support from the Grant PID2023-149516NB-I00/ AEI/10.13039/501100011033/ FEDER, UE

**5.)**	Thank you for stating the following in your Competing Interests section: N/A. 
Please complete your Competing Interests on the online submission form to state any Competing Interests. If you have no competing interests, please state ""The authors have declared that no competing interests exist."", as detailed online in our guide for authors at <http://journals.plos.org/plosone/s/submit-now> 
 This information should be included in your cover letter; we will change the online submission form on your behalf.

Done.

[Note: HTML markup is below. Please do not edit.]

**Reviewers' comments:**

**Reviewer's Responses to Questions**

**Comments to the Author**

**1.)**	Is the manuscript technically sound, and do the data support the conclusions?

The manuscript must describe a technically sound piece of scientific research with data that supports the conclusions. Experiments must have been conducted rigorously, with appropriate controls, replication, and sample sizes. The conclusions must be drawn appropriately based on the data presented.

Reviewer #1: Yes

Reviewer #2: Partly

**Reply:**	We have included in the introduction a new paragraph explaining which are the main objectives of this work, including another one briefly anticipating the results obtained. Thus, we have written:

*“* ***…*** *This study examines the statistical properties of four primary air pollutants in Ulaanbaatar, Mongolia. This work has two main objectives: first, to determine if time trends are present in the data to show potential decreasing trends in its temporal evolution. Second, we use a specific model named fractional integration, with which, due to its characteristics, we can determine the degree of persistence in the data with a single parameter (the order of integration) and whether shocks in the series will have transitory or permanent effects. Our results indicate that the four pollutants examined are mean reverting with shocks having transitory effects, and significant negative trends are found in the cases of SO_2_ and PM_2.5_. …“.*

**2.)**	Has the statistical analysis been performed appropriately and rigorously?

Reviewer #1: Yes

Reviewer #2: I Don't Know

**Reply:** Thank you.

**3.)**	Have the authors made all data underlying the findings in their manuscript fully available?

The [PLOS Data policy](http://track.editorialmanager.com/CL0/http:%2F%2Fwww.plosone.org%2Fstatic%2Fpolicies.action%23sharing/1/010f0194fa9f5c1e-2582ea45-8cb9-479c-94f0-12df57419e82-000000/_hHZtCeCDM72qQFe8065RdjuMJv4Czk_QUoZlYZXBXQ=198) requires authors to make all data underlying the findings described in their manuscript fully available without restriction, with rare exception (please refer to the Data Availability Statement in the manuscript PDF file). The data should be provided as part of the manuscript or its supporting information, or deposited to a public repository. For example, in addition to summary statistics, the data points behind means, medians and variance measures should be available. If there are restrictions on publicly sharing data—e.g. participant privacy or use of data from a third party—those must be specified.

Reviewer #1: No

Reviewer #2: Yes

**Reply:** Thanks. We have included the following comment in the Data Description and Methodological section:

“ *… The air pollution pollutants in Ulaanbaatar, Mongolia, were officially measured by the National Agency for Meteorology and Environmental Monitoring (NAMEM) at 19 monitoring locations. The daily data used in this study was obtained from NAMEM or a state-run network. According to the World Health Organization (WHO), the major air pollutants include PM_2.5_, PM_10_, O₃, NO₂, SO₂, and CO. In Mongolia, air quality monitoring follows WHO guidelines; however, not all pollutants are consistently measured. Some monitoring stations record only three pollutants—PM_10_, SO₂, and NO₂—while others measure four, depending on equipment availability. Only a few monitoring stations measure all six major pollutants. The selected monitoring station, UB-2, is located in the center of Ulaanbaatar city. This site measures four pollutants: PM_10_, PM_2.5_, SO₂, and NO₂. Therefore, due to this limitation and justification, these four variables were used in the analysis. …*”.

**4.)**	Is the manuscript presented in an intelligible fashion and written in standard English?

PLOS ONE does not copyedit accepted manuscripts, so the language in submitted articles must be clear, correct, and unambiguous. Any typographical or grammatical errors should be corrected at revision, so please note any specific errors here.

Reviewer #1: Yes

Reviewer #2: Yes

**Reply:** Thanks. We have revised the manuscript several times to avoid typos and grammatical errors.

**Reviewer #1:**

**1.)**	Why only 4 pollutants are taken? This should be addressed properly as by EPA norms, 6 pollutants should be present in any kind of analysis.

**Reply:** Thanks for the comment.

Our study focuses on primary pollutants in Mongolia, where coal combustion is the primary source of air pollution due to its widespread use in heating systems. Other studies similar to ours either focus on analysing the spatiotemporal characteristics of primary air pollutants or on assessing air pollutants focused on these four pollutants. For instance, Zeng et al. (2019) evaluated the effectiveness of energy policies using spatial econometric methods, focusing on PM_10_, PM_2.5_, and SO_2_. Tian et al. (2023) examined major air pollutants' spatiotemporal and sectoral distribution and their drivers, selecting NO_x_, SO_2_, and dust as key pollutants. Similarly, Priya & Sathya (2019) focused on SO_2_, NO_2_, and PM in India, while Lau et al. (2024) analysed PM_2.5_ and NO_2_ using citywide air monitoring data from New York, USA. Other studies have adopted geospatial analysis to assess pollution trends. Sidharthan et al. (2025) monitored SO_2_, NO_2_, and PM using GIS modelling in India. Singh et al. (2023) analysed monthly data from five different monitoring stations in India, covering NO_2_, SO_2_, and PM₁₀ from 2011 to 2020. Li et al. (2020) investigated the spatiotemporal characteristics of PM_2.5_, PM_10_, SO_2_, and NO_2_ across 11 stations in China. Other relevant studies include Mulgeta et al. (2024), who analysed the spatial distribution of PM_2.5_ and PM_10_ in Addis Ababa, Ethiopia. Li et al. (2015) focused on identifying the main contributors to air pollution in Beijing, specifically analysing SO₂ levels. Sharma et al. (2019) examined air pollution trends across various geographical locations in India, considering SO_2_, NO_x_, and PM_2.5._ Additionally, Razy-Yanuv et al. (2022) updated previous marine air pollution estimations by analysing emissions of NO_x_, SO_x_, and PM_2.5_.

The above comments have now been added at the beginning of the Literature Review section.

Also, air quality monitoring follows WHO guidelines; however, not all pollutants are consistently measured in Mongolia. Some monitoring stations record only three pollutants—PM_10_, SO_2_, and NO_2_ —while others measure four, depending on equipment availability. Only a few stations monitor all six major pollutants. The selected monitoring site for this study, located in central Ulaanbaatar, measures four pollutants: PM_10_, PM_2.5_, SO_2_, and NO_2_. Consequently, these four variables were used in the analysis.

Dealing with this point, we have added the following comment:

“ *… The air pollution pollutants in Ulaanbaatar, Mongolia, were officially measured by the National Agency for Meteorology and Environmental Monitoring (NAMEM) at 19 monitoring locations. The daily data used in this study was obtained from NAMEM or a state-run network. According to the World Health Organization (WHO), the major air pollutants include PM_2.5_, PM_10_, O₃, NO₂, SO₂, and CO. In Mongolia, air quality monitoring follows WHO guidelines; however, not all pollutants are consistently measured. Some monitoring stations record only three pollutants—PM_10_, SO₂, and NO₂—while others measure four, depending on equipment availability. Only a few monitoring stations measure all six major pollutants. The selected monitoring station, UB-2, is located in the center of Ulaanbaatar city. This site measures four pollutants: PM_10_, PM_2.5_, SO₂, and NO₂. Therefore, due to this limitation and justification, these four variables were used in the analysis. …*”.

**2.)**	Limited literature review- Literature Review has to be given in a more detailed way. To make it convenient for others to read papers with concise structures, please summarize some related papers, for example:
<https://ieeexplore.ieee.org/abstract/document/10363642>
<https://link.springer.com/article/10.1007/s11869-015-0369-9>
<https://ieeexplore.ieee.org/abstract/document/10529960>
<https://sensors.myu-group.co.jp/sm_pdf/SM3362.pdf>
<https://link.springer.com/article/10.1007/s11270-024-07012-9>
<https://ieeexplore.ieee.org/document/10706023>
<https://ieeexplore.ieee.org/document/10806566>

**Reply:**	Many thanks for letting us know about all these interesting papers. Accordingly, we have read them and introduced some of them in the literature review, which seems now much more complete. In addition, we have also included the papers mentioned in point 1) above. Dealing specifically with the above papers, we have added the following:

*“ … A Simple Linear Regression (SLR) model was used by Rabuan et al. (2023) to evaluate the accuracy of five low-cost air quality sensors against a particulate reference analyzer. Their findings suggest that low-cost sensors are unreliable for accurately measuring air quality in indoor environments. Various studies have also applied machine learning (ML) and artificial neural network (ANN) models. Borah et al. (2023) introduced a location-invariant air pollution prediction model with strong geographic generalizability, integrating light gradient-boosted regression (GBR) within a ML framework. Experiments on diverse datasets demonstrated superior performance to standard forecasting methods, such as recurrent neural networks and transformers. An interpretability analysis identified key factors influencing air pollution levels and revealed geographical patterns of high pollutant concentrations. Furthermore, Borah et al. (2024) predicted the concentrations of six major air pollutants using ML and deep learning techniques, while Patra et al. (2016) applied an ANN model to predict dust concentration in India’s deepest opencast copper mines, achieving strong agreement between observed and predicted data. …”.*

**3.)**	Comparison with the state of the art literature is not present, newer publications should be compared

**Reply:**	We have expanded our discussion section by comparing findings with newer studies on air pollution, such as focusing on comparing air pollution trends with recent studies in similar countries from the perspective of locations and how our study contributes new insights compared to previous works. We have added the following comments in the discussion section:

“ *… In comparing our findings with the latest studies on similarly polluted countries, Morawska et al. (2021) investigated the number, frequency, and duration of pollution episodes. Their study analysed baseline air pollution trends in 100 cities, focusing on daily PM_2.5_ concentrations. The results classified Delhi and Beijing into Group 1, characterised by a positive R_PE,norm_ norm trend, indicating a decline in overall air pollution—while the event rate continued to rise. This suggests that although PM_2.5_ concentrations are decreasing, the frequency and severity of pollution episodes are increasing, consistent with our findings. Furthermore, using data on spatiotemporal variations and trends, Wang et al. (2021) and Maji & Sarkar (2020) observed a decreasing trend in SO_2_ concentrations. However, NO_2_ trends showed minimal improvement (−0.45 ± 2.0 μg/m³/year), significantly lower than the reduction observed for SO_2_ and PM_2.5_ in China. These findings align with the results of our study, further supporting the air quality trends observed.*

*Kazakhstan has geographical conditions similar to Mongolia's and exhibits significant air pollution levels. Kerimray et al. (2020) reported that PM_10_ and NO_2_ concentrations in Kazakhstan exceed WHO air quality guidelines by 2 and 6.8 times, respectively, in other countries with comparable geography and pollution levels. Their study emphasised the need for continued research and monitoring to understand air quality trends better. However, we identified a gap in defining future air pollution trends in Kazakhstan. The methodology used in this study could serve as a valuable resource for future research in this region to establish clearer projections. …”.*

**Minor comments are as follows:**

**1.)**	Inconsistent Terminology: The manuscript uses different terms for similar concepts which could confuse readers.

**Reply:**	Thanks.

Line 73:	PM2.5 levels changed to PM2.5 concentration

Line 94:	SO2 levels changed to SO2 concentration

Line 111:	PM levels changed to PM concentrations

Line 303:	Emission of NO_x_ changed to the concentration of NO_x_

Line 305:	NO_x_ emission changed to NO_x_ concentration; PM emission changed to PM concentration

Line 306-Line 308:	All the “emission” words changed to “concentration”.

**2.)**	Insufficient Justification for air pollutants.

**Reply:**	Thanks for the comment. We have provided a justification for selecting four air pollutants in response to Major Comment 1. In addition, we would like to elaborate on the source-specific rationale for this selection. The primary sources of air pollution in Ulaanbaatar, Mongolia, are household heating systems, power plants (both reliant on coal combustion), and transportation. Numerous studies have examined the sources of air pollution in Ulaanbaatar and have consistently identified household emissions as the dominant contributor, followed by power plant emissions and transportation-related pollution. The following references support this conclusion:

Batmunkh, T., Kim, Y. J., Jung, J. S., Park, K., & Tumendemberel, B. (2013). Chemical characteristics of fine particulate matters measured during severe winter haze events in Ulaanbaatar, Mongolia. Journal of the Air & Waste Management Association, 63(6), 659-670. https://doi.org/10.1080/10962247.2013.776997

Davy, P. K., Gunchin, G., Markwitz, A., Trompetter, W. J., Barry, B. J., Shagjjamba, D., & Lodoysamba, S. (2011). Air particulate matter pollution in Ulaanbaatar, Mongolia: determination of composition, source contributions and source locations. Atmospheric Pollution Research, 2(2), 126-137. DOI:10.5094/APR.2011.017

Government of Mongolia, Swiss Agency for Development and Cooperation SDC, UNICEF. (2023). Impact of air pollution on maternal and child health project. p10. Available online: https://www.unicef.org/mongolia/media/5071/file/Impact%20of%20Air%20Pollution%20on%20Maternal%20and%20Child%20Health%20Project%20(2018-2023).pdf (accessed on 18 February 2025)

Guttikunda, S. K., Lodoysamba, S., Bulgansaikhan, B., & Dashdondog, B. (2013). Particulate pollution in Ulaanbaatar, Mongolia. Air Quality, Atmosphere & Health, 6, 589-601. DOI: 10.1007/s11869-013-0198-7

The Air Pollution Reducing Department of Capital City (APRD). (2023). Source Emissions Inventory Annual Report 2021. p46-50 Available online: https://aprd.ub.gov.mn/index.php?newsid=729 (accessed on 18 February 2025)

The Air Pollution Reducing Department of Capital City (APRD)., Japan International Cooperation Agency (JICA). (2017), Capacity Development Project for Air Pollution Control in Ulaanbaatar City Phase 2 in Mongolia Final Report. p147-159 Available online: https://openjicareport.jica.go.jp/pdf/12289310.pdf (accessed on 18 February 2025)

Zinicovscaia, I., Narmandakh, J., Yushin, N., Peshkova, A., Chaligava, O., Tsendsuren, T. O., ... & Tsogbadrakh, T. (2024). Assessment of Air Pollution in Ulaanbaatar Using the Moss Bag Technique. Archives of Environmental Contamination and Toxicology, 86(2), 152-164. https://doi.org/10.1007/s00244-024-01050-4

See the new paragraph on the Introduction (page 2) explaining this point further.

**3.)**	Lack of Future Work Discussion: The manuscript does not outline potential future research directions or applications of the findings, which could enhance its impact.

**Reply:**	Thanks for the comment. We have extended the final paragraph in the conclusions that reads now as follows:

***“ …*** *Firstly, robustness checking of the results presented can be elaborated by using other parametric or even semiparametric methods. Some initial investigation, based on Sowell’s (1992) maximum likelihood method in the time domain or the semiparametric log-periodogram approach of Geweke and Porter-Hudak (GPH, 1983; Robinson, 1995; Shimotsu and Phillips, 2002), produced results that though quantitatively might differ in some cases, qualitatively were very similar, supporting the hypothesis of fractional integration in all cases. Also, from a methodological viewpoint, non-linear structures can be considered. The linear trends used in this work can be replaced by non-linear polynomials in time such as those based on Chebyshev polynomials in time and used in Cuestas and Gil-Alana (2016) or by Fourier functions (Gil-Alana and Yaya, 2021) or even neural networks (Yaya et al., 2021). It would be interesting to determine if the same conclusions as those reported in this work hold under these different assumptions. From an empirical viewpoint, the analysis can be extended to other big cities all over the world. Work in these directions is now in progress. …”.*

**4.)**	Figures and Tables Not Referenced: Several figures and tables are mentioned but not adequately referenced in the text, making it hard for readers to follow the results.

**Reply:** Thanks. All figures and tables are now referenced.

**7.)**	Inconsistent Data Presentation: The presentation of data in figures and tables lacks uniformity, making it harder to interpret the results effectively.

**Reply:** Thanks. We have better explained the meaning of tables and figures in the revised version of the paper.

**Other minor changes**

**Title page:**	Comments from the Editor and two anonymous reviewers are gratefully acknowledged.

***.)**	We have corrected several typos and grammatical errors all over the manuscript.

Line 158:	We have replaced Naveen (2017) by Naveen and Anu (2017).

Tables 3 and 5: We have explained in an endnote that --- means lack of statistical significance.

**References**

Batmunkh, T., Kim, Y. J., Jung, J. S., Park, K., & Tumendemberel, B. (2013). Chemical characteristics of fine particulate matters measured during severe winter haze events in Ulaanbaatar, Mongolia. *Journal of the Air & Waste Management Association*, *63*(6), 659-670. <https://doi.org/10.1080/10962247.2013.776997>

Cuestas J.C. & Gil-Alana, L.A. (2016). A Non-Linear Approach with Long Range Dependence Based on Chebyshev Polynomials. Studies in Nonlinear Dynamics and Econometrics, 23, 445–468.

Davy, P. K., Gunchin, G., Markwitz, A., Trompetter, W. J., Barry, B. J., Shagjjamba, D., & Lodoysamba, S. (2011). Air particulate matter pollution in Ulaanbaatar, Mongolia: determination of composition, source contributions and source locations. *Atmospheric Pollution Research*, *2*(2), 126-137. DOI:10.5094/APR.2011.017

Ethiopia. *Stochastic Environmental Research and Risk Assessment*, 1-19.

Geweke, J. and S. Porter-Hudak (1983), The estimation and applicatons of long memory time series models, Journal of Time Series Analysis 4(4), 221-238.

Gil-Alana, L.A. & Yaya, O. (2021). Testing fractional unit roots with non-linear smooth break approximations using Fourier functions. Journal of Applied Statistics, 48, 13-15, 2542-2559.

Guttikunda, S. K., Lodoysamba, S., Bulgansaikhan, B., & Dashdondog, B. (2013). Particulate pollution in Ulaanbaatar, Mongolia. *Air Quality, Atmosphere & Health*, *6*, 589-601. DOI: 10.1007/s11869-013-0198-7

Kerimray, A., Assanov, D., Kenessov, B., & Karaca, F. (2020). Trends and health impacts of major urban air pollutants in Kazakhstan. *Journal of the Air & Waste Management Association*, *70*(11), 1148-1164. <https://doi.org/10.1080/10962247.2020.1813837>

Lau, K., Guo, J., Miao, Y., Ross, Z., Riley, K. W., Wang, S., ... & Perera, F. (2024). Major air pollution and climate policies in NYC and trends in NYC air quality 1998–2021. *Frontiers in Public Health*, *12*, 1474534.

Li, S., Feng, K., & Li, M. (2017). Identifying the main contributors of air pollution in Beijing. *Journal of Cleaner Production*, *163*, S359-S365.

Li, L., Zhao, Z., Wang, H., Wang, Y., Liu, N., Li, X., & Ma, Y. (2020). Concentrations of four major air pollutants among ecological functional zones in Shenyang, Northeast China. *Atmosphere*, *11*(10), 1070.

Maji, K. J., & Sarkar, C. (2020). Spatio-temporal variations and trends of major air pollutants in China during 2015–2018. *Environmental Science and Pollution Research*, *27*, 33792-33808. <https://doi.org/10.1007/s11356-020-09646-8>

Morawska, L., Zhu, T., Liu, N., Torkmahalleh, M. A., de Fatima Andrade, M., Barratt, B., ... & Ye, C. (2021). The state of science on severe air pollution episodes: Quantitative and qualitative analysis. *Environment international*, *156*, 106732. <https://doi.org/10.1016/j.envint.2021.106732>

Mulgeta, D., Gotu, B., Temesgen, S., Belina, M., Likassa, H. T., & Tsegaye, D. (2024). Statistical Analysis of Spatial Distribution of Ambient Air Pollution in Addis Ababa,

Naveen, V.; Anu, N. (2017). Time series analysis to forecast air quality indices in Thiruvananthapuram District, Kerala, India. Journal of Engineering Research and Application 7(6), 66-84. https://doi: 10.9790/9622-0706036684

Priya, S. & Sathya, P. (2019, March). Statistical analysis of air pollutants in ambient air, reality of sensors and corrective measures in India. In *2019 Innovations in Power and Advanced Computing Technologies (i-PACT)* (Vol. 1, pp. 1-6). IEEE.

Razy-Yanuv, E., Barak, Y., Noam, O., & Madar, D. (2022). Marine air pollution in Israel: extent, proposed mitigation targets, benefits and feasibility. *Atmosphere*, *13*(2), 241.

Robinson, P.M. (1995), Gaussian Semiparametric Estimation of Long Range Dependence, Annals of Statistics 23 (5) 1630-1661. https://doi.org/10.1214/aos/1176324317

Sharma, R., Kumar, R., Sharma, D. K., Son, L. H., Priyadarshini, I., Pham, B. T., ... & Rai, S. (2019). Inferring air pollution from air quality index by different geographical areas: case study in India. *Air Quality, Atmosphere & Health*, *12*, 1347-1357.

# Shimotsu, K. and P.C.B. Phillips (2002), Pooled Log Periodogram Regression, Journal of Time Series Analysis 23(1), 57-93.

Sidharthan, K., Gandhimathi, A., & Akshayaa, M. (2025). Spatial variation of air pollutants by using GIS modelling. Global NEST Journal 27, Issue 3.

Singh, A., Deep, A., Pandey, C., & Nandan, H. (2023). Comparative study of gaseous pollutants for major cities in foothills of Garhwal Himalaya of Uttarakhand. MAUSAM, *74*(1), 57-72.

Sowell, F. (1992), Maximum likelihood estimation of stationary univariate fractionally integrated time series models, Journal of Econometrics 53 (1-3), 165-188.

The Air Pollution Reducing Department of Capital City (APRD). (2023). Source Emissions Inventory Annual Report 2021. p46-50 Available online: <https://aprd.ub.gov.mn/index.php?newsid=729> (accessed on 18 February 2025)

The Air Pollution Reducing Department of Capital City (APRD)., Japan International Cooperation Agency (JICA). (2017), Capacity Development Project for Air Pollution Control in Ulaanbaatar City Phase 2 in Mongolia Final Report. p147-159 Available online: <https://openjicareport.jica.go.jp/pdf/12289310.pdf> (accessed on 18 February 2025)

Tian, Y., He, C., Yang, L., Yi, J., Ke, B., Mu, H., ... & Hong, S. (2023). Spatiotemporal Dynamic Correlation Characteristics and Driving Factors of Major Air Pollutant Emissions in China. *Atmosphere*, *14*(1), 130.

Wang, Y., Ali, M. A., Bilal, M., Qiu, Z., Mhawish, A., Almazroui, M., ... & Haque, M. N. (2021). Identification of NO2 and SO2 pollution hotspots and sources in Jiangsu Province of China. *Remote Sensing*, *13*(18), 3742. <https://doi.org/10.3390/rs13183742>

Yaya, O., Ogbonna, A.E., Gil-Alana, L.A. & Furuoka, F. (2021). A new unit root analysis for testing hysteresis in unemployment. Oxford Bulletin of Economics and Statistics, 83(4), 960-981.

Zeng, J., Liu, T., Feiock, R., & Li, F. (2019). The impacts of China's provincial energy policies on major air pollutants: A spatial econometric analysis. *Energy Policy*, *132*,

Zinicovscaia, I., Narmandakh, J., Yushin, N., Peshkova, A., Chaligava, O., Tsendsuren, T. O., ... & Tsogbadrakh, T. (2024). Assessment of Air Pollution in Ulaanbaatar Using the Moss Bag Technique. *Archives of Environmental Contamination and Toxicology*, *86*(2), 152-164. <https://doi.org/10.1007/s00244-024-01050-4>

**Reviewer #2:**

This manuscript describes the behaviour of air pollutants over an extended period of time and concludes that at least two of them have significantly reduced their levels. It is of mostly local relevance, but the fact that it focuses on an area that it is not well described yet, it becomes of general interest.

However, I feel the manuscript right now is not sharing important information regarding the origin and quality of the data set presented. It is analyzing secondary data without showing proper validation. An extended analysis of the location of sampling sites is also needed. Additionally, I recommend extending the conclusions to infer about nearby locations (or similar locations worldwide). Thus, the manuscript will have more global relevance instead of just being useful for local readers.
 **Reply:** Thanks for the comments. Dealing with the dataset, we have provided a much more detailed analysis in the new version, which also describes the data source and provides a clear justification for the selection of the four major pollutants. We acknowledge the reviewer's concern regarding the validation and origin of the dataset. As our study relies on air quality data from the National Agency for Meteorology and Environmental Monitoring (NAMEM), we are not directly involved in the data collection, including instrument specifications and validation. Therefore, we are unable to provide detailed information on these aspects. However, NAMEM follows national and WHO guidelines for air quality monitoring, ensuring standardised data collection.

We have added the following comment in the Data description and Methodology section:

“ *… The air pollution pollutants in Ulaanbaatar, Mongolia, were officially measured by the National Agency for Meteorology and Environmental Monitoring (NAMEM) at 19 monitoring locations. The daily data used in this study was obtained from NAMEM or a state-run network. According to the World Health Organization (WHO), the major air pollutants include PM_2.5_, PM_10_, O₃, NO₂, SO₂, and CO. In Mongolia, air quality monitoring follows WHO guidelines; however, not all pollutants are consistently measured. Some monitoring stations record only three pollutants—PM_10_, SO₂, and NO₂—while others measure four, depending on equipment availability. Only a few monitoring stations measure all six major pollutants. The selected monitoring station, UB-2, is located in the center of Ulaanbaatar city. This site measures four pollutants: PM_10_, PM_2.5_, SO₂, and NO₂. Therefore, due to this limitation and justification, these four variables were used in the analysis. …*”.

In response to the reviewer's recommendations, we have extended the conclusion from a methodological perspective as a future work discussion and expanded the discussion section by incorporating comparisons with recent studies on air pollution. Specifically, we have included a comparison of air pollution trends in similar countries, analysing findings from the perspective of location, environmental conditions, and high pollution levels (Kazakhstan, China and India).

In particular, dealing with this specific point, we have added the following comments:

“ *… In comparing our findings with the latest studies on similarly polluted countries, Morawska et al. (2021) investigated the number, frequency, and duration of pollution episodes. Their study analysed baseline air pollution trends in 100 cities, focusing on daily PM_2.5_ concentrations. The results classified Delhi and Beijing into Group 1, characterised by a positive R_PE,norm_ norm trend, indicating a decline in overall air pollution—while the event rate continued to rise. This suggests that although PM_2.5_ concentrations are decreasing, the frequency and severity of pollution episodes are increasing, consistent with our findings. Furthermore, using data on spatiotemporal variations and trends, Wang et al. (2021) and Maji & Sarkar (2020) observed a decreasing trend in SO_2_ concentrations. However, NO_2_ trends showed minimal improvement (−0.45 ± 2.0 μg/m³/year), significantly lower than the reduction observed for SO_2_ and PM_2.5_ in China. These findings align with the results of our study, further supporting the air quality trends observed.*

*Kazakhstan has geographical conditions similar to Mongolia's and exhibits significant air pollution levels. Kerimray et al. (2020) reported that PM_10_ and NO_2_ concentrations in Kazakhstan exceed WHO air quality guidelines by 2 and 6.8 times, respectively, in other countries with comparable geography and pollution levels. Their study emphasised the need for continued research and monitoring to understand air quality trends better. However, we identified a gap in defining future air pollution trends in Kazakhstan. The methodology used in this study could serve as a valuable resource for future research in this region to establish clearer projections. …”.*

Some **minor comments** are included:

**1.)**	Please include a description of how the data was generated in the first place, it is important to locate the reader on the type of study presented.

**Reply:**	Thank you very much for the information. The data description and methodology section is divided into three subsections now. The first subsection, **Data Collection and Monitoring Method** provides details on how the data was generated and the source of data used in this study. See the comment below:

***Data collection and air quality monitoring***

*The National Agency for Meteorology and Environmental Monitoring (NAMEM) at 19 monitoring locations officially measured the air pollution pollutants in UB, Mongolia. The daily data used in this study was obtained from NAMEM. In Mongolia, air quality monitoring follows WHO guidelines; however, not all pollutants are consistently measured. Some monitoring stations record only three pollutants—PM_10_, SO₂, and NO₂—while others measure four, depending on equipment availability. Only a few monitoring stations measure all six major pollutants. Due to these limitations, We selected a single monitoring site for representing the UB. This site, located in central Ulaanbaatar, measures four pollutants: PM_10_, PM_2.5_, SO₂, and NO₂.*

**2.)**	Section 2 of the manuscript is interesting, but it needs to clarify what kind of methods were used in each study. Only then does the comparison make more sense. I would put a special focus on the sample collection/recording methods.

**Reply:** Thanks for the comment. We have improved this section by briefly explaining the different methodologies and by including additional references which are related to our work. The whole section reads now as follows:

*“ This study focuses on primary pollutants in Mongolia, where coal combustion is the primary source of air pollution due to its widespread use in heating systems. Other studies similar to ours either focus on analysing the spatiotemporal characteristics of primary air pollutants or on assessing air pollutants focused on these four pollutants. For instance, Zeng et al. (2019) evaluated the effectiveness of energy policies using spatial econometric methods, focusing on PM_10_, PM_2.5_, and SO_2_. Tian et al. (2023) examined major air pollutants' spatiotemporal and sectoral distribution and their drivers, selecting NO_x_, SO_2_, and dust as key pollutants. Similarly, Priya & Sathya (2019) focused on SO_2_, NO_2_, and PM in India, while Lau et al. (2024) analysed PM_2.5_ and NO_2_ using citywide air monitoring data from New York, USA. Other studies have adopted geospatial analysis to assess pollution trends. Sidharthan et al. (2025) monitored SO_2_, NO_2_, and PM using GIS modelling in India. Singh et al. (2023) analysed monthly data from five different monitoring stations in India, covering NO_2_, SO_2_, and PM₁₀ from 2011 to 2020. Li et al. (2020) investigated the spatiotemporal characteristics of PM_2.5_, PM_10_, SO_2_, and NO_2_ across 11 stations in China. Other relevant studies include Mulgeta et al. (2024), who analysed the spatial distribution of PM_2.5_ and PM_10_ in Addis Ababa, Ethiopia. Li et al. (2015) focused on identifying the main contributors to air pollution in Beijing, specifically analysing SO₂ levels. Sharma et al. (2019) examined air pollution trends across various geographical locations in India, considering SO_2_, NO_x_, and PM_2.5._ Additionally, Razy-Yanuv et al. (2022) updated previous marine air pollution estimations by analysing emissions of NO_x_, SO_x_, and PM_2.5_.*

*Focussing on the air pollution in Mongolia, Luvsan et al. (2012) concluded that air pollution, specifically SO_2_ concentration, has worsened due to rapid urbanisation and industrialisation since the mid-1990s. SO_2_ concentrations were obtained from the Central Laboratory of Environmental Monitoring (CLEM) under NAMHEM, which operates Mongolia's air quality monitoring network. This study used SO_2_ data and meteorological parameters with at least 70% data completeness, recorded from 14 sites—including the capital and provincial centres—between January 1, 1996, and December 31, 2009. Pollution is found to be particularly severe in urban areas, near steel industry sites, and during winter. It is noticeable that in UB, SO_2_ concentrations increase with decreasing wind speed and temperature, as well as with increasing relative humidity. Meteorological parameters and emissions from industrial sources and gers to the north of the city seem to influence the dispersion of SO_2_ in UB. Suriya et al. (2022) focused on PM_10_ and PM_2.5,_ which are the primary pollutants. However, they estimate the air quality index (AQI) for major pollutants such as PM_2.5_, PM_10_, SO_2_, NO_2_, CO, and O_3_. The daily monitoring data was obtained from the Mongolian Ministry of Nature, Environment, and Tourism; the hourly monitoring data for PM_2.5_ and PM_10_ were obtained from the OpenAQ website. The meteorological data was obtained from the National Oceanic and Atmospheric Administration.*

*According to researchers, the PM_2.5_/PM_10_ ratio has decreased yearly, with the highest concentrations in winter and the lowest in summer in UB. Daily PM concentration showed a bimodal pattern: it decreased during the day and slightly increased in the afternoon due to temperature inversion. PM concentrations were significantly higher during the heating season, indicating coal-fired heating as the main cause of air pollution in UB.*

*Byambaa et al. (2019) identified sources in UB city by characterising polycyclic aromatic hydrocarbons in total suspended particles. Researchers collected samples from five locations using a high-volume air sampler for 3-24 hours. Concentrations varied across different sites and seasons, with the highest in the ger area during winter. The main pollution source in the city centre during winter was vehicle emissions, while other sites showed mixed contributions from coal, petroleum, and other biomass combustion. Soyol-Erdene et al. (2021) suggested some recommendations for further research to improve air quality monitoring. One key recommendation in the paper is the establishment of more air quality monitoring sites and the enhancement of air quality investigations which this study addresses.*

*From the methodological side, several papers have employed fractional integration in air quality analysis. Thus, for example,* [*Gil-Alana et al. (2020)*](https://journals.ametsoc.org/view/journals/apme/59/8/jamcD200040.xml) *used a fractionally integrated framework to analyze U.S. air quality using datasets retrieved from the EPA database. This allowed fractional differentiation degrees for stationary I(0) series, offering more flexibility in the dynamic data specification. Additionally, Gil-Alana et al. (2023) investigated the time trends and persistence of PM_2.5_ in 20 megacities. They employed techniques based on long-range dependence or long memory, with a focus once again on fractional integration models using daily average data taken from the World AQI. The advantage of using a long-memory fractionally integrated framework in analysing air quality time series data lies in its ability to capture complex and persistent patterns in the data. This methodology allows for a more nuanced understanding of the dynamics of air quality variables, such as particulate matter pollution, by considering fractional values in the degree of differentiation. Other papers using a similar methodology in the analysis of air pollution include Zhongfei et al. (2016) in four Chinese cities, Caporale et al. (2021) in ten European capitals and Gil-Alana et al. (2020) in the case of London. Alternative methodological approaches such as AutoRegressive Integrated Moving Average (ARIMA) have been employed in the analysis of Indian air quality data by Naveen and Anu (2017), Kulkarni et al. (2018) and Gopu et al. (2021), while Guan-Yu et al. (2022) and Mei et al. (2023) use a hybrid deep learning/Kriging model and a new complex-network-based model respectively.* *A Simple Linear Regression (SLR) model was used by Rabuan et al. (2023) to evaluate the accuracy of five low-cost air quality sensors against a particulate reference analyzer. Their findings suggest that low-cost sensors are unreliable for accurately measuring air quality in indoor environments. Various studies have also applied machine learning (ML) and artificial neural network (ANN) models. Borah et al. (2023) introduced a location-invariant air pollution prediction model with strong geographic generalizability, integrating light gradient-boosted regression (GBR) within a ML framework. Experiments on diverse datasets demonstrated superior performance to standard forecasting methods, such as recurrent neural networks and transformers. An interpretability analysis identified key factors influencing air pollution levels and revealed geographical patterns of high pollutant concentrations. Furthermore, Borah et al. (2024) predicted the concentrations of six major air pollutants using ML and deep learning techniques, while Patra et al. (2016) applied an ANN model to predict dust concentration in India’s deepest opencast copper mines, achieving strong agreement between observed and predicted data.*

**3.)**	Also, please describe briefly what fractional integration is.

**Reply:**	The methodological section has been improved by including more details of the approach employed in the paper. Thus, we have added the following:

*“ … Dealing with the methodology, we use a particular type of long memory model widely used in the context of environmental studies and denominated fractional integration. The idea that is behind this concept is that the number of differences required in a time series to render it stationary I(0) may be a non-integer positive value. In other words, a process {x(t), t = 0, ±1, …} is said to be fractionally integrated or integrated of order d, and denoted as I(d) if it can be represented as:*

*(1 – B)^d^ x(t) = u(t), t = 0, ±1, …	(1)*

*where B is the backshift operator, such that B^k^x(t) = x(t-k) and u(t) is I(0) or integrated of order 0, which means that it is a second order (or covariance) stationary process with a spectral density function that is bounded and positive at all frequencies. Within this category, the simplest process is the white noise**, characterized by displaying a zero mean, a constant variance and no autocorrelation, though it also permits serial correlation like that of the stationary Autoregressive Moving Average (ARMA) processes.*

*The magnitude of the differencing parameter d is crucial to determine if exogenous shocks in the series have transitory or permanent effects. Thus, if that value is smaller than 1, shocks are expected to be transitory and the recovery is faster the lower the value of d is. On the other hand, values of d equal to or superior to 1 indicate a lack of mean reversion and thus permanency of shocks. Moreover, the fact that d is a real value permits us to consider a wide range of alternatives, including among others,*

*i)	short memory or I(0) processes, if d = 0,*

*ii)	stationary long memory or I(d) processes, if 0 < d < 0.5,*

*iii)	nonstationary though mean reverting I(d) processes, if 0.5 ≤ d < 1,*

*iv)	unit roots or I(1) processes, if d = 1, and*

*v)	explosive patterns, if d > 1.*

*Of particular interest in the present work are the process in iii) since being nonstationary, they still display transitory shocks though with long lasting effects. In the empirical work conducted in the following section, we are also interested in the potential presence of trends in the data. Thus, we assume now that x(t) in (1) are the errors in a regression that includes an intercept and a linear time trend, i.e.,*

*y(t)	=	α	+	β t	+	x(t), t =	1, 2, …,	(2)*

*where y(t) is the series corresponding to the observed data; α and β* *are the unknown constant and trend respectively, and x(t) follows Equation (1).*

*The estimation is conducted via the log-likelihood function, following a procedure developed in Robinson (1994) and widely used when analyzing univariate time series data. Its functional form can be found in Gil-Alana and Robinson (1997), and applications in environmental science using this approach are, among others, Zhongfei et al. (2016), Gil-Alana and Solarin (2018), Gil-Alana, Yaya and Carmona (2020), Caporale, Gil-Alana and Carmona (2021) and Gil-Alana, Bermejo and del Rio (2023).*

*This method is a testing procedure. The null hypothesis is:*

*H_o_: d = d_o_,	(3)*

*which is tested in the model given by Eqs. (1) and (2) for any real value d_o_. It is based on the Lagrange Multiplier (LM) principle and it has a standard normal null and local limit distributions. This allows us to consider a confidence band for the non-rejection values of d. In addition, this standard limit behaviour holds whether or not deterministic terms (like those in (2)) are included in the model, and it is supposed to be the most efficient method in the Pitman sense against local alternatives. (See, Robinson, 1994). …”*

**4.)**	What is the origin of the data shown in table 1? Please describe. Where was it recorded?

**Reply:** Data source has now been included in the Data description and Methodology section. See the comments above.

**5.)**	By using what kind of instruments? Was it validated?

**Reply:**	The air pollution pollutants in Ulaanbaatar, Mongolia, were officially measured by the National Agency for Meteorology and Environmental Monitoring (NAMEM) at 19 monitoring locations. The validated daily data used in this study was obtained from NAMEM’s station or a state-run network.

**6.)**	Define what white noise errors are

**Reply:** We have explained that a white noise process is a process with zero mean, constant variance and uncorrelated components.

**7.)**	Line 69: is there any evidence of that? Or is it just the authors opinion?

**Reply:** Line 69's conclusion is from the official report and other research studies. Here are the references:

Batmunkh, T., Kim, Y. J., Jung, J. S., Park, K., & Tumendemberel, B. (2013). Chemical characteristics of fine particulate matters measured during severe winter haze events in Ulaanbaatar, Mongolia. Journal of the Air & Waste Management Association, 63(6), 659-670. https://doi.org/10.1080/10962247.2013.776997

Davy, P. K., Gunchin, G., Markwitz, A., Trompetter, W. J., Barry, B. J., Shagjjamba, D., & Lodoysamba, S. (2011). Air particulate matter pollution in Ulaanbaatar, Mongolia: determination of composition, source contributions and source locations. Atmospheric Pollution Research, 2(2), 126-137. DOI:10.5094/APR.2011.017

Government of Mongolia, Swiss Agency for Development and Cooperation SDC, UNICEF. (2023). Impact of air pollution on maternal and child health project. p10. Available online: https://www.unicef.org/mongolia/media/5071/file/Impact%20of%20Air%20Pollution%20on%20Maternal%20and%20Child%20Health%20Project%20(2018-2023).pdf (accessed on 18 February 2025)

Guttikunda, S. K., Lodoysamba, S., Bulgansaikhan, B., & Dashdondog, B. (2013). Particulate pollution in Ulaanbaatar, Mongolia. Air Quality, Atmosphere & Health, 6, 589-601. DOI: 10.1007/s11869-013-0198-7

The Air Pollution Reducing Department of Capital City (APRD). (2023). Source Emissions Inventory Annual Report 2021. p46-50 Available online: https://aprd.ub.gov.mn/index.php?newsid=729 (accessed on 18 February 2025)

The Air Pollution Reducing Department of Capital City (APRD)., Japan International Cooperation Agency (JICA). (2017), Capacity Development Project for Air Pollution Control in Ulaanbaatar City Phase 2 in Mongolia Final Report. p147-159 Available online: https://openjicareport.jica.go.jp/pdf/12289310.pdf (accessed on 18 February 2025)

Zinicovscaia, I., Narmandakh, J., Yushin, N., Peshkova, A., Chaligava, O., Tsendsuren, T. O., ... & Tsogbadrakh, T. (2024). Assessment of Air Pollution in Ulaanbaatar Using the Moss Bag Technique. Archives of Environmental Contamination and Toxicology, 86(2), 152-164. <https://doi.org/10.1007/s00244-024-01050-4>

They are all now mentioned in the Introduction section, on page 3.

**8.)**	Line 75: are these values measured by a state-run network? Or were they published elsewhere?

**Reply:** A state-run network measures all the data referenced by the National Statistic Committee, and they publish an annual report on their website.

**Other minor changes**

**Title page:**	Comments from the Editor and two anonymous reviewers are gratefully acknowledged.

***.)**	We have corrected several typos and grammatical errors all over the manuscript.

Line 158:	We have replaced Naveen (2017) by Naveen and Anu (2017).

Tables 3 and 5: We have explained in an endnote that --- means lack of statistical significance.

**References**

Batmunkh, T., Kim, Y. J., Jung, J. S., Park, K., & Tumendemberel, B. (2013). Chemical characteristics of fine particulate matters measured during severe winter haze events in Ulaanbaatar, Mongolia. Journal of the Air & Waste Management Association, 63(6), 659-670. https://doi.org/10.1080/10962247.2013.776997

Borah, J., Kumar, S., Kumar, N., Nadzir, M. S. M., Cayetano, M. G., Ghayvat, H., ... & Kumar, N. (2023). AiCareBreath: IoT enabled location invariant novel unified model for predicting air pollutants to avoid related respiratory disease. *IEEE Internet of Things Journal*. DOI: [10.1109/JIOT.2023.3342872](https://doi.org/10.1109/JIOT.2023.3342872)

Borah, J., Nadzir, M. S. M., Cayetano, M. G., Majumdar, S., Ghayvat, H., & Srivastava, G. (2024). AiCareAir: Hybrid-Ensemble Internet of Things Sensing Unit Model for Air Pollutant Control. IEEE Sensors Journal. DOI: [10.1109/JSEN.2024.3397735](https://doi.org/10.1109/JSEN.2024.3397735)

Byambaa, B., Yang, L., Matsuki, A., Nagato, E. G., Gankhuyag, K., Chuluunpurev, B., ... & Hayakawa, K. (2019). Sources and characteristics of polycyclic aromatic hydrocarbons in ambient total suspended particles in Ulaanbaatar City, Mongolia. *International Journal of Environmental Research and Public Health*, *16*(3), 442. <https://doi.org/10.3390/ijerph16030442>

Caporale, G.M., L.A. Gil-Alana and N. Carmona (2021), Particular Matter PM10. Persistence and trends in eight European capitals, Air Quality, Atmosphere and Health 14, 1097-1102. <https://doi.org/10.1007/s11869-021-01002-0>

Davy, P. K., Gunchin, G., Markwitz, A., Trompetter, W. J., Barry, B. J., Shagjjamba, D., & Lodoysamba, S. (2011). Air particulate matter pollution in Ulaanbaatar, Mongolia: determination of composition, source contributions and source locations. Atmospheric Pollution Research, 2(2), 126-137. DOI:10.5094/APR.2011.017

Gil-Alana, L.A. and S. Solarin (2018), Have US environmental policies been effective in the reduction of US emissions? A new approach using fractional integration, Atmospheric Pollution Research 9, 1, 53-60. <https://doi.org/10.1016/j.apr.2017.06.008>

Gil-Alana, L.A., L. Bermejo and M. del Rio (2023), Time trends and persistence in PM25 in 20 megacities. Evidence for the time period 2018-2020, Environmental Sciences and Pollution Research 30,3 3, 5603-5620. <https://doi.org/10.1007/s11356-022-22512-z>

Gil-Alana, L.A. and P.M. Robinson, (1997). Testing of unit root and other nonstationary hypotheses in macroeconomic time series. Journal of Econometrics, 80(2), 241-268. doi:10.1016/s0304-4076(97)00038-9

Gil-Alana, L.A., O. Yaya and N. Carmona (2020), Air quality in London. Evidence of persistence, seasonality and trends, Theoretical and Applied Climatology 142, 103-115. <https://doi.org/10.1007/s00704-020-03305-1>

Gopu, P.: Panda, R.R.; Nagwani, N.K. (2021). Time Series Analysis Using ARIMA Model for Air Pollution Prediction in Hyderabad City of India. In: Reddy, V.S., Prasad, V.K., Wang, J., Reddy, K.T.V. (eds) Soft Computing and Signal Processing. Advances in Intelligent Systems and Computing, vol 1325. Springer, Singapore. https://doi.org/10.1007/978-981-33-6912-2_5

Government of Mongolia, Swiss Agency for Development and Cooperation SDC, UNICEF. (2023). Impact of air pollution on maternal and child health project. p10. Available online: https://www.unicef.org/mongolia/media/5071/file/Impact%20of%20Air%20Pollution%20on%20Maternal%20and%20Child%20Health%20Project%20(2018-2023).pdf (accessed on 18 February 2025)

Guan-Yu, L.; Yi-Ming, L.; Chuen-Jinn, T.; Chia-Ying, L. (2022). Spatial-temporal characterization of air pollutants using a hybrid deep learning/Kriging model incorporated with a weather normalization technique, Atmospheric Environment 289, 119304, 352-2310, https://doi.org/10.1016/j.atmosenv.2022.119304.

Guttikunda, S. K., Lodoysamba, S., Bulgansaikhan, B., & Dashdondog, B. (2013). Particulate pollution in Ulaanbaatar, Mongolia. Air Quality, Atmosphere & Health, 6, 589-601. DOI: 10.1007/s11869-013-0198-7

Kerimray, A., Assanov, D., Kenessov, B., & Karaca, F. (2020). Trends and health impacts of major urban air pollutants in Kazakhstan. *Journal of the Air & Waste Management Association*, *70*(11), 1148-1164. <https://doi.org/10.1080/10962247.2020.1813837>

Kulkarni, G.E.; Muley, A.A.; Deshmukh, N.K.; Bhalchandra, P.U. (2018)*.* Autoregressive integrated moving average time series model for forecasting air pollution in Nanded city,

Maharashtra, India. Modeling Earth Systems and Environment 4, 1435–1444. <https://doi.org/10.1007/s40808-018-0493-2>

Li, S., Feng, K., & Li, M. (2017). Identifying the main contributors of air pollution in Beijing. *Journal of Cleaner Production*, *163*, S359-S365.

Li, L., Zhao, Z., Wang, H., Wang, Y., Liu, N., Li, X., & Ma, Y. (2020). Concentrations of four major air pollutants among ecological functional zones in Shenyang, Northeast China. *Atmosphere*, *11*(10), 1070.

Luvsan, M. E., Shie, R. H., Purevdorj, T., Badarch, L., Baldorj, B., & Chan, C. C. (2012). The influence of emission sources and meteorological conditions on SO2 pollution in Mongolia. *Atmospheric Environment*, *61*, 542-549. <https://doi.org/10.1016/j.atmosenv.2012.07.044>

Maji, K. J., & Sarkar, C. (2020). Spatio-temporal variations and trends of major air pollutants in China during 2015–2018. *Environmental Science and Pollution Research*, *27*, 33792-33808. <https://doi.org/10.1007/s11356-020-09646-8>

Mei, C.; Yongxu C.; Hongyu, Z.; Youshuai, W.; Yue, X. (2023). Analysis of pollutants transport in heavy air pollution processes using a new complex-network-based model, Atmospheric Environment 292, 19395, <https://doi.org/10.1016/j.atmosenv.2022.119395>.

Morawska, L., Zhu, T., Liu, N., Torkmahalleh, M. A., de Fatima Andrade, M., Barratt, B., ... & Ye, C. (2021). The state of science on severe air pollution episodes: Quantitative and qualitative analysis. *Environment international*, *156*, 106732. <https://doi.org/10.1016/j.envint.2021.106732>

Mulgeta, D., Gotu, B., Temesgen, S., Belina, M., Likassa, H. T., & Tsegaye, D. (2024). Statistical Analysis of Spatial Distribution of Ambient Air Pollution in Addis Ababa,

Naveen, V.; Anu, N. (2017). Time series analysis to forecast air quality indices in Thiruvananthapuram District, Kerala, India. Journal of Engineering Research and Application 7(6), 66-84. https://doi: 10.9790/9622-0706036684

Priya, S. & Sathya, P. (2019, March). Statistical analysis of air pollutants in ambient air, reality of sensors and corrective measures in India. In *2019 Innovations in Power and Advanced Computing Technologies (i-PACT)* (Vol. 1, pp. 1-6). IEEE.

Rabuan, U., Mohd Nadzir, M. S., Abdullah Sham, S. Z., Izzati Wan Shaiful Bahri, S. B., Borah, J., Majumdar, S., ... & Mohd Yunus, N. H. (2023). Evaluations of Low-cost Air Quality Sensors for Particulate Matter (PM 2.5) under Indoor and Outdoor Conditions. *Sensors & Materials*, *35*. <https://doi.org/10.18494/SAM4393>

Razy-Yanuv, E., Barak, Y., Noam, O., & Madar, D. (2022). Marine air pollution in Israel: extent, proposed mitigation targets, benefits and feasibility. *Atmosphere*, *13*(2), 241.

Robinson, P.M. (1994). Efficient tests of nonstationary hypotheses. Journal of the American Statistical Association, 89, 1420-1437. <https://doi.org/10.1080/01621459.1994.10476881>

Sharma, R., Kumar, R., Sharma, D. K., Son, L. H., Priyadarshini, I., Pham, B. T., ... & Rai, S. (2019). Inferring air pollution from air quality index by different geographical areas: case study in India. *Air Quality, Atmosphere & Health*, *12*, 1347-1357.

Sidharthan, K., Gandhimathi, A., & Akshayaa, M. (2025). Spatial variation of air pollutants by using GIS modelling. Global NEST Journal 27, Issue 3.

Soyol-Erdene, T. O., Ganbat, G., & Baldorj, B. (2021). Urban air quality studies in Mongolia: pollution characteristics and future research needs. *Aerosol and Air Quality Research*, *21*(12), 210163. <https://doi.org/10.4209/aaqr.210163>

Suriya, Natsagdorj, N., Aorigele, Zhou, H., & Sachurila. (2022). Spatiotemporal variation in air pollution characteristics and influencing factors in Ulaanbaatar from 2016 to 2019. *Atmosphere*, *13*(6), 990. <https://doi.org/10.3390/atmos13060990>

The Air Pollution Reducing Department of Capital City (APRD). (2023). Source Emissions Inventory Annual Report 2021. p46-50 Available online: https://aprd.ub.gov.mn/index.php?newsid=729 (accessed on 18 February 2025)

The Air Pollution Reducing Department of Capital City (APRD)., Japan International Cooperation Agency (JICA). (2017), Capacity Development Project for Air Pollution Control in Ulaanbaatar City Phase 2 in Mongolia Final Report. p147-159 Available online: https://openjicareport.jica.go.jp/pdf/12289310.pdf (accessed on 18 February 2025)

# Tian, Y, He, C., Yang, L., Yi, L., Ke, B., Mu, H., Tu, P., Ye, Z., Hong, S. (2023), Spatiotemporal Dynamic Correlation Characteristics and Driving Factors of Major Air Pollutant Emissions in China, Atmosphere 14(1), 130; <https://doi.org/10.3390/atmos14010130>

Wang, Y., Ali, M. A., Bilal, M., Qiu, Z., Mhawish, A., Almazroui, M., ... & Haque, M. N. (2021). Identification of NO2 and SO2 pollution hotspots and sources in Jiangsu Province of China. *Remote Sensing*, *13*(18), 3742. <https://doi.org/10.3390/rs13183742>

Zeng, J., Liu, T., Feiock, R., & Li, F. (2019). The impacts of China's provincial energy policies on major air pollutants: A spatial econometric analysis. *Energy Policy*, *132*,

Zhongfei C.; Barros C.P.; Gil-Alana, L.A. (2016) The persistence of air pollution in four mega-cities of China, Habitat International 56, 103-108, <https://doi.org/10.1016/j.habitatint.2016.05.004>. <https://doi.org/10.1016/j.habitatint.2016.05.004>

Zinicovscaia, I., Narmandakh, J., Yushin, N., Peshkova, A., Chaligava, O., Tsendsuren, T. O., ... & Tsogbadrakh, T. (2024). Assessment of Air Pollution in Ulaanbaatar Using the Moss Bag Technique. Archives of Environmental Contamination and Toxicology, 86(2), 152-164. <https://doi.org/10.1007/s00244-024-01050-4>

***.)**	 PLOS authors have the option to publish the peer review history of their article ([what does this mean?](https://track.editorialmanager.com/CL0/https:%2F%2Fjournals.plos.org%2Fplosone%2Fs%2Feditorial-and-peer-review-process%23loc-peer-review-history/1/010f0194fa9f5c1e-2582ea45-8cb9-479c-94f0-12df57419e82-000000/frJYdMHC9MAdVGBUNsSzRwEWByy1fmnBlTTN5LsDjvI=198)). If published, this will include your full peer review and any attached files.


If you choose “no”, your identity will remain anonymous but your review may still be made public.


**Do you want your identity to be public for this peer review?** For information about this choice, including consent withdrawal, please see our [Privacy Policy](https://track.editorialmanager.com/CL0/https:%2F%2Fwww.plos.org%2Fprivacy-policy/1/010f0194fa9f5c1e-2582ea45-8cb9-479c-94f0-12df57419e82-000000/Pytc9YV7hB9_6lYVlHXXgG9SYvDc7ekHebxQfUhn80g=198).

Reviewer #1: No

Reviewer #2: No

Many thanks for all these valuable comments.

*In compliance with data protection regulations, you may request that we remove your personal registration details at any time.* [*(Remove my information/details)*](https://track.editorialmanager.com/CL0/https:%2F%2Fwww.editorialmanager.com%2Fpone%2Flogin.asp%3Fa=r/1/010f0194fa9f5c1e-2582ea45-8cb9-479c-94f0-12df57419e82-000000/mC2EIHEi76fjixVnkX-MyCwBMJ6CORVjsTeszuwDl78=198)*. Please contact the publication office if you have any questions.*
